# BcSUN1, a *B. cinerea* SUN-Family Protein, Is Involved in Virulence

**DOI:** 10.3389/fmicb.2017.00035

**Published:** 2017-01-20

**Authors:** Alicia Pérez-Hernández, Mario González, Celedonio González, Jan A. L. van Kan, Nélida Brito

**Affiliations:** ^1^Departamento de Bioquímica, Microbiología, Biología Celular y Genética, Universidad de La Laguna (ULL)La Laguna, Spain; ^2^Laboratory of Phytopathology, Department of Plant Sciences, Wageningen University and Research Centre (WUR)Wageningen, Netherlands

**Keywords:** *Botrytis cinerea*, BcSUN1, glycoprotein, secretome, cell wall, virulence

## Abstract

BcSUN1 is a glycoprotein secreted by *Botrytis cinerea*, an important plant pathogen that causes severe losses in agriculture worldwide. In this work, the role of BcSUN1 in different aspects of the *B. cinerea* biology was studied by phenotypic analysis of *Bcsun1* knockout strains. We identified BcSUN1 as the only member of the Group-I SUN family of proteins encoded in the *B. cinerea* genome, which is expressed both in axenic culture and during infection. BcSUN1 is also weakly attached to the cellular surface and is involved in maintaining the structure of the cell wall and/or the extracellular matrix. Disruption of the *Bcsun1* gene produces different cell surface alterations affecting the production of reproductive structures and adhesion to plant surface, therefore reducing *B. cinerea* virulence. BcSUN1 is the first member of the SUN family reported to be involved in the pathogenesis of a filamentous fungus.

## Introduction

*Botrytis cinerea* has been considered the second most important plant pathogenic fungus according to its economic/scientific importance (Dean et al., 2012). During infection of plant tissues, the fungus secretes hundreds of proteins to the extracellular medium, although just a few of them have been reported, by analysis of the corresponding mutants, to have a significant contribution to *B. cinerea* virulence (González et al., 2015).

BcSUN1 is a member of the β-glucosidase SUN family that has been experimentally identified as a component of the *B. cinerea* secretome (Espino et al., 2010; González et al., 2014). The *Bcsun1* gene encodes a protein of 471 amino acids that contains a signal peptide for secretion, as well as several Ser/Thr-rich regions that are potentially hyper-*O*-glycosylated (González et al., 2012). Oligosaccharides with mannose α1-2 and/or α1-3 bonds, but not mannose α1-6 bonds, were experimentally confirmed to be present in the BcSUN1 protein (González et al., 2014). In addition, the culture medium from a *B. cinerea* strain overexpressing BcSUN1 showed an enhanced capacity to elicit plant defenses, as compared with the wild type strain (González et al., 2014), suggesting that BcSUN1 may be recognized by the plant immune system.

The β-glucosidase SUN family of proteins (Pfam PF03856; Interpro IPR005556) were first described in *Saccharomyces cerevisiae* and have been identified only in ascomycetes (Firon et al., 2007). In spite of their annotation, β-glucosidase activity has been described only for AfSUN1 from *Aspergillus fumigatus* and SUN41 from *Candida albicans*, assigned to the new Glycosyl Hydrolase family GH132 (Gastebois et al., 2013). Structurally, proteins of this family are classified into two groups (Firon et al., 2007). Group-I members show a well conserved *C*-terminal region of 258 amino acids corresponding to the SUN domain, which comprises four putative Fe-binding cysteine residues (Cys-X_5_-Cys-X_3_-Cys-X_24_-Cys) (Mouassite et al., 2000a), and a less conserved *N*-terminal region, which contains a signal peptide and a low complexity region rich in Ser and Thr residues. Group-II proteins harbor a degenerate SUN domain, with multiple amino acid insertions in the Cys-rich motif and a shorter *N*-terminal region (Firon et al., 2007; Gastebois et al., 2013). Members of Group-II are all related to the *S. cerevisiae* YMR244W protein (Firon et al., 2007).

Group-I members have been extensively studied in yeast, and diverse biological functions have been attributed to them. The four prototypical SUN proteins of *S. cerevisiae* namely SIM1, UTH1, NCA3 and SUN4 (Mouassite et al., 2000a,b) are involved in different cell functions. SIM1 plays an important role in the regulation of DNA replication (Dahmann et al., 1995), although when overproduced from a multicopy plasmid, SIM1 also functioned as an extracellular suppressor of mutations in the PAG1 and CBK1 genes involved in cellular morphogenesis (Du and Novick, 2002). UTH1 was first identified in a screening for *S. cerevisiae* mutants with increased stress resistance and longer life spans (Kennedy et al., 1995) and shows a dual localization: in mitochondria, where it is involved in mitochondrial biogenesis and autophagy (Camougrand et al., 2000) and in the cell wall, where it seems to play a role in determining the β-d-glucan/chitin composition (Ritch et al., 2010). The third member of the family, NCA3, is involved in the maturation of transcripts encoding two components of the ATP-synthase complex in mitochondria (Pelissier et al., 1995). Finally, SUN4 was isolated as a soluble cell wall protein (Cappellaro et al., 1998) and is involved in cell septation (Mouassite et al., 2000a). Similarly to UTH1, SUN4 has also been found both in the cell wall and in mitochondria (Cappellaro et al., 1998; Mouassite et al., 2000a). Recently, UTH1, SIM1 and SUN4 have also been described as secreted proteins, and their production was affected by the level of oxygen (Kuznetsov et al., 2013). In *C. albicans*, on the contrary, only two members of the SUN family have been identified, SUN41 and SUN42, both of which are involved in remodeling the cell wall and are essential for cell separation (Firon et al., 2007; Hiller et al., 2011).

The SUN family has been poorly studied in filamentous fungi. To our knowledge, these proteins have been experimentally analyzed only in *A. fumigatus* (Gastebois et al., 2013), a saprophytic fungus typically found in soil and decaying organic matter, which can also cause aspergillosis in humans (Kwon-Chung and Sugui, 2013), and in *Ustilaginoidea virens*, the causal agent of rice false smut disease (Yu et al., 2015). AfSUN1 from *A. fumigatus*, a member of Group-I, has been reported to be involved in fungal morphogenesis (Gastebois et al., 2013) and the Group-II protein, UvSUN2 from *U. virens*, has been proposed to be involved in cell wall biogenesis and response to stress (Yu et al., 2015).

In this work we report the disruption of the *Bcsun1* gene in *B. cinerea* and the phenotypic characterization of the mutant. We show that BcSUN1 plays a key role in fungal morphogenesis and is required for full virulence.

## Materials and Methdos

### Strains and Growth Conditions

*Botrytis cinerea* strains used in this work were B05.10 (Quidde et al., 1999), a wild type strain, and B05.10-BcSUN1, which expresses a tagged version of the BcSUN1 protein under the control of the *OliC* promoter (González et al., 2014). These were kept as conidial suspensions in 15% glycerol at -80°C for long storage, and were maintained on 3% malt extract agar (MEA, Oxoid, UK) plates for routine use. Fungal cultures were routinely incubated at 22°C. *B. cinerea* conidia were prepared as described by Benito et al. (1998) from cultures on tomato-plates (25% homogenized tomato fruits, 1.5% agar, pH 5.5). Unless otherwise indicated, fungal strains were grown on YGG medium [0.5% yeast extract, 2% glucose, and 0.3% Gamborg’s B5 (Duchefa Biochemie, The Netherlands)], supplemented with 1.5% agar and 100 μg/ml hygromycin or nourseothricin when required. As minimal medium, GB5 (0.3% Gamborg’s B5, 1% glucose, 10 mM KH_2_PO_4_) was used. To examine production of the extracellular matrix (ECM) under the microscope, conidia were germinated in PDB medium (0.1% Potato dextrose broth, Duchefa Biocheme, The Netherlands). To analyze different extracellular protein fractions, conidia were germinated in YGG-L medium (0.3% Gamborg’s B5, 0.36% glucose, 10 mM KH_2_PO_4_, 10 mM MES (Sigma Aldrich, USA), 0.5% yeast extract, pH 5.5).

*Nicotiana tabacum* var. Havana, *Solanum lycopersicum* var. moneymaker and *Phaseolus vulgaris* plants were maintained in a growth chamber at 22°C, 70% humidity with a light/dark cycle of 14 h light/10 h dark. When tobacco seedlings were required, seeds were sterilized as explained before (González et al., 2014) and incubated 2 days at 4°C in darkness to break dormancy before germination on solid MS medium [0.5% Murashige and Skoog (Duchefa Biochemie, The Netherlands), 0.8% agar, pH 5.7] for 1 week.

### Quantitative Real-Time PCR (Q-RT-PCR)

Mycelia for RNA extraction were prepared as described elsewhere (ten Have et al., 2010). Briefly, 7 × 10^6^ conidia/ml were germinated for 12 h in 250 ml of GB5 medium containing 2 mM sucrose instead of glucose and a dialysis bag with 30 ml of a 50% (w/v) kiwi, tomato or strawberry fruit extract (made in the same medium). As a control, standard GB5 medium was used and no dialysis bag was added. For the *in planta* expression studies, RNA was isolated from infected tomato leaves as described before (Brito et al., 2006).

One microgram of total RNA was used as template for cDNA synthesis using the iScript cDNA Synthesis Kit (Bio-Rad, USA), according to the manufacturer’s instructions. Q-RT-PCR reactions were performed in an iCycler iQ thermal cycle (Bio-Rad, USA) with the iQ SYBR Green Supermix (Bio-Rad, USA) and the primers listed in Supplementary Table S1. In order to normalize the expression levels, the *B. cinerea actA* gene was used as an internal reference. One of the two primers for each transcript spanned over an exon–exon junction on the cDNA to avoid amplification from contaminant genomic DNA. The relative mRNA amounts were calculated by the ΔΔCt method from the mean of three independent determinations of the threshold cycle (Ct), and the control sample (ungerminated conidia) was used as calibrator (Schmittgen and Livak, 2008). Deviation from the mean for each sample was calculated from the standard deviation (SD) in the ΔΔCt value using the expression 2^(ΔΔCt±SD)^.

### *Bcsun1* Gene Disruption

The replacement cassette was constructed by Overlap Extension PCR as described by Nelson and Fitch (2011), with some modifications, and the strategy is outlined in Supplementary Figure S1. Genomic DNA from *B. cinerea* was extracted using the PUREGENE DNA Purification Kit (Qiagen, USA) and primers (Supplementary Table S1) were from Biolegio (Nijmegen, The Netherlands). All PCR products and restriction endonuclease digestion fragments were purified with *PCR Clean-up Kit* and *NucleoSpin Gel kit* (MACHEREY-NAGEL, Germany), respectively. The two fragments homologous to the target gene were amplified with *GoTaqG2 DNA Polymerase* (Promega, USA) and primer pairs 6.5.1/6.5.3 for the 5′-flanking region, and 6.3.1/6.3.3 for 3′-flanking region. The hygromycin resistance cassette was obtained by digestion of the pLOB7 vector (Zhang et al., 2011) with *Eco*RI and *Hind*III. The three fragments were fused by PCR using the *Expand High Fidelity Enzyme Mix* (Roche, Switzerland). The resultant gene replacement cassette was purified, checked by double digestions with *EcoR*I-*Sac*II and *Hind*III-*Sac*II, and used to transform *B. cinerea* protoplasts (van Kan et al., 1997). Homokaryons were purified from the transformants obtained and analyzed by Southern blot and PCR (Supplementary Figure S1). Two independent knockout lines (Δ*Bcsun1.1* and Δ*Bcsun1.2*) were used in all experiments. Since the phenotypes of both independent mutants were identical, only the results of Δ*Bcsun1.1* are shown in most figures for simplicity.

### Phenotypic Analysis

Fungal sensitivity to a range of compounds was assayed determining the growth rate on YGG plates supplemented with one of the following chemicals: 30 mM H_2_O_2_ (Foret, Spain), 0.005% congo red (CR; Sigma–Aldrich, USA), 0.02% SDS, 0.05% Calcofluor white (CW; Sigma–Aldrich, USA), 1 M sorbitol, or 0.4% boric acid (BA; Merck-Millipore, Germany). Colony radius was measured every 24 h during 3 days.

Sensitivity of mycelium to protoplast-forming enzymes was analyzed by treating young mycelium with Lysing Enzymes from *Trichoderma harzianum* (Sigma–Aldrich, USA). Conidia were germinated for 16 h in YGG medium, washed three times with KC buffer (0.6 M KCl, 50 mM CaCl_2_), and incubated for up to 4 h in 7.5 mg/ml of the enzyme mix in KC buffer. Samples were taken every 30 min and protoplasts were counted using a haemocytometer.

The capacity of the mycelium to retain water was calculated by comparing the fresh weight of the mycelium after filtration with the dry weight after being completely dried. Fungal strains were grown for 3 days in 20 ml of YGG medium in the dark, without shaking. Cultures were then filtered and the mycelia were allowed to drain for 30 min at room temperature and weighed (fresh weight) and then dried at 60°C to a constant weight (dry weight). The water retention capacity was calculated as the ratio of the amount of water retained (fresh weight minus dry weight) to the corresponding dry weight.

Pathogenicity tests were performed by inoculating bean, tomato or tobacco leaves with either agar plugs containing young mycelium (0.2-cm YGG-agar cubes) or conidia suspensions (5-μl droplets of 2.5 × 10^5^ conidia/ml in TGGK solution (60 mM KH_2_PO_4_, 10 mM glycine, 0.01% Tween 20, 100 mM glucose). The inoculated leaves were incubated at 20°C under conditions of high humidity on water-soaked filter paper in closed containers. At different time points after inoculation, lesions on leaves were photographed and their radii were measured. Quantitative results are presented as the percentage of expanding lesions per total number of inoculation spots, and the rate of increase of lesion size (in cm/day) of the expanding lesions. Adhesion of *B. cinerea* to plant surfaces was assayed as described by González et al. (2013). The number of conidia on infected leaves was estimated at 10 days post-inoculation using squares of infected leaf (4 × 4 cm). Conidia were released by vortexing in ddH_2_O for 20 s and then quantified as described elsewhere (González et al., 2013).

To compare the amount of ECM around hyphae, the fungus was grown for 3 days in 500 μl of PDB medium (inoculated with 5 × 10^5^ conidia), on glass slides in high-humidity conditions. The medium was then aspirated and the mycelium was completely overlaid with several drops of black India ink, covered with a coverslip, and observed under the microscope (Olympus BX-50). Aggregation of conidia was observed under the microscope at 2 h after inoculation in YGG medium. To analyze the production of conidiophores and infection cushions, agar plugs with young mycelium were laid on glass slides and incubated 10 days under high-humidity before observation under the microscope. To quantify the number of germ tubes per conidium and their rate of ramification, conidia were germinated for 16 h in YGG medium and observed under the fluorescence microscope after staining with CW for easier visualization. Staining was done by incubation in a CW solution (0.05% CW in 3.75% KOH) for 5 min, washing twice with 15% KOH, and resuspension in 20 μl of 3.75% KOH/4.35% glycerol. The microscope (Olympus BX-50) was equipped with a U-MWIB filter.

Production of reactive oxygen species (ROS) by fungal strains was assayed according to Viefhues et al. (2015) with minor modifications. Briefly, YGG-agar solid medium was overlaid with cellophane, inoculated with mycelium plugs, incubated for 4 days, and finally used to harvest 6 mg of fresh mycelium that was then placed at the bottom of a well in a microtiter (96 wells) plate. Then 250 μl of a 3,3′-Diaminobenzidine (DAB) solution (0.5 mg/ml DAB in 100 mM citric acid, pH 3.7) were added to cover the mycelium, and the plate was incubated for 1.5 h in the dark and visually evaluated. Positive and negative controls were done as in Viefhues et al. (2015). Production of ROS during infection was assayed in tobacco leaves inoculated with 5-μl droplets of a conidial suspension (5 × 10^5^ conidia/ml in TGGK solution). 40 h after inoculation, leaf disks with the infected area in its center were cut and vacuum infiltrated for 1 h with 1 mg/ml DAB, pH 3.8. To visualize the ROS stain, disks were then boiled in ethanol for 5 min to eliminate chlorophyll, and photographed. Quantification of ROS from the images obtained was done with the software Fiji (Schindelin et al., 2012) and is expressed as the percentage of brown pixels detected in a circumference of constant area around the infection point.

### Extracellular Protein Fractions and BcSUN1 Localization

The strain B05.10-BcSUN1 was grown for 16 h with shaking (160 rpm) in YGG-L medium supplemented with 25 μg/ml nourseothricin and 4 μg/ml pepstatin-A (Sigma–Aldrich, USA), inoculated with 3 × 10^6^ conidia/ml. Three protein fractions were recovered from the culture: (i) the extracellular proteins were recovered from culture filtrates; (ii) proteins non-covalently attached to the fungal cells were isolated incubating the collected mycelium with 300 mM NaCl (15 ml/g mycelium) for 15 min and subsequent filtration, recovering the NaCl-solubilized proteins; and (iii) the remaining mycelial proteins were obtained by incubation of the NaCl-treated mycelium with Laemmli sample buffer (Laemmli, 1970) (0.1 mg mycelium in 100 μl buffer). Extracellular and NaCl-solubilized proteins were precipitated with methanol-chloroform according to Wessel and Flügge (1984) and the pellets were also resuspended in Laemmli sample buffer. The three protein preparations were fractionated by SDS-PAGE, electroblotted onto nitrocellulose membranes (Whatman Protran BA 85), and BcSUN1 was detected with mouse anti-*c*-*myc* antibodies (Sigma–Aldrich, USA; 1:5000 dilution) in combination with anti-mouse IgG conjugated to Horseradish peroxidase (Sigma–Aldrich, USA; 1:3000 dilution) as the secondary antibody. The peroxidase signal was detected with Immobilon Western Chemiluminescent HRP Substrate (Merk-Millipore, Germany) and the intensity of the bands was measured with the software Quantity One (BioRad, USA).

### Statistical Analysis

Statistical analysis was carried out with SPSS 17 (IBM). Statistical significance tests used were either the *T*-test, in those cases with a normal distribution (analyzed with the Kolmogorov–Smirnov test), or the Mann-Whitney test, if sample distribution was not normal. Asterisks indicate a statistically significant difference with the control (wild type strain) (*p* = 0.05).

## Results

### BcSUN1 is the Only Member of the Group-I of SUN Family in *B. cinerea*

BcSUN1 (Bcin06g06040.1, from the *B. cinerea* protein database in EnsemblFungi^1^; van Kan et al., 2016) is a highly glycosylated protein of 48 kDa, initially identified as a component of the *B. cinerea* early secretome (Espino et al., 2010) and also present in the glycosecretome (González et al., 2014). The alignment of its sequence with the four *S. cerevisiae* proteins that belong to Group-I of the SUN family (Supplementary Figure S2) showed an overall identity ranging from 28.1% for NCA3 to 38.6% for SIM1 (**Table 1**), while sequence conservation was higher for the *C*-terminal region containing the SUN domain, with amino acid sequence identities ranging from 41 to 46%. Analysis of the hydropathic profiles (Supplementary Figure S3) also established a good similarity to Group-I proteins, especially at the *C*-terminus. BcSUN1 is especially similar to AfSUN1 from *A. fumigatus* (43.5% of amino acid identity), the only member of Group-I experimentally studied in filamentous fungi (Gastebois et al., 2013).

**Table 1:** Amino acid sequence identity and similarity of BcSUN1 to *S. cerevisiae* proteins in Group-I of the SUN family.

	Protein		*C*-terminal region	
	Identity (%)	Similarity (%)	Identity (%)	Similarity (%)
SIM1	38.6	52.9	41.6	58.0
UTH1	36.6	47.9	46.2	57.3
NCA3	28.1	41.0	43.0	58.3
SUN4	33.7	48.6	43.4	57.5

*Identity and similarity were computed with EMBOSS Needle from alignments of the entire sequences or the C-terminal regions only. Uniprot accession numbers for the *S. cerevisiae* proteins are as follows: P40472 (SIM1), P36135 (UTH1), P32493 (NCA3) and P53616 (SUN4).*

A BLAST-P search (Altschul et al., 1997) in the *B. cinerea* genome^1^ with BcSUN1 as the query sequence did not identify additional homologues belonging to Group-I. However, the search revealed an additional SUN family member (gene Bcin07g06600.1) showing 53% amino acid identity both with the hypothetical protein YMR244W from *S. cerevisiae* and β-glucosidase Adg3 from *Schizosaccharomyces pombe*, both classified as members of the Group-II of the SUN family (Firon et al., 2007). This gene encodes a protein of 530 amino acids containing a signal sequence for secretion, according to SignalP 4.1, and a degenerate SUN domain, with multiple amino acid insertions in the N-terminal Cys-rich motif (data not shown).

### BcSUN1 is Expressed Both in Axenic Culture and *In planta*

**FIGURE 1:**
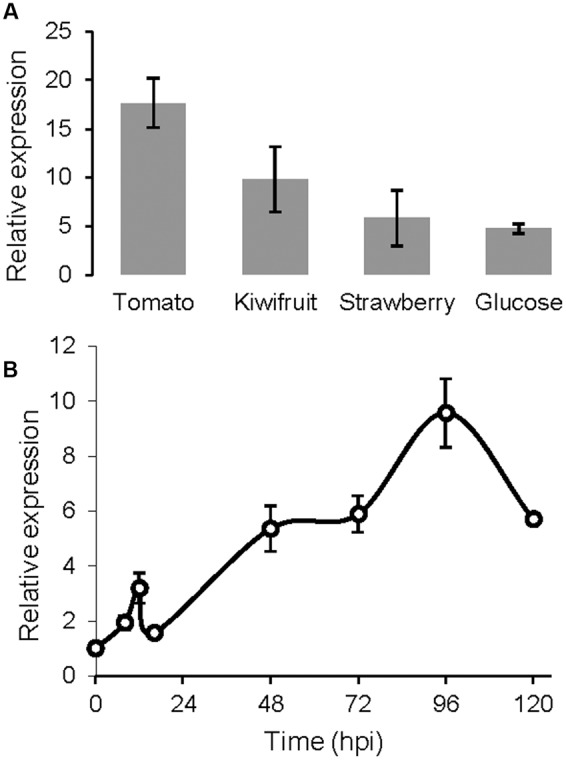
**Levels of *Bcsun1* mRNA in the wild type strain B05.10 under different growth conditions. (A)** Levels of *Bcsun1* mRNA in the wild type strain B05.10 grown for 12 h in liquid GB5 medium containing, as indicated, a dialysis bag with 50% (w/v) kiwi, tomato or strawberry fruit extract, or supplemented with 1% glucose as carbon source. **(B)** Levels of *Bcsun1* mRNA in tomato leaves infected with *B. cinerea* B05.10 at various hours after inoculation (hpi). In all cases data are relative to the mRNA levels in ungerminated conidia. Results are expressed as mean ± SD of 3 technical replicates.

BcSUN1 has been detected in the culture medium, as a secreted protein, very early after conidial germination and also at 4 days after inoculation on YGG (Espino et al., 2010; González et al., 2014). The expression of the *Bcsun1* gene was studied in more detail in this work by Q-RT-PCR. In axenic culture, *Bcsun1* mRNA levels increased significantly in every condition tested at 12 h post inoculation, as compared with the expression in non-germinated conidia (**Figure 1A**). The level of induction was higher when plant extracts were included in the medium than in a chemically defined medium with glucose as the only carbon source. The levels of *Bcsun1* mRNA also increased during the infection of tomato leaves with *B. cinerea* (**Figure 1B**). A slight induction (2.5 times in comparison to ungerminated conidia) was observed in the early phase of infection (up to 12 h) and then, after a slight drop, expression levels increased up to 96 h post inoculation (**Figure 1B**). These results suggest a role for BcSUN1 during infection, especially at late stages when the lesions become necrotic.

### BcSUN1 is Involved in Maintaining Cell Wall Integrity

**FIGURE 2:**
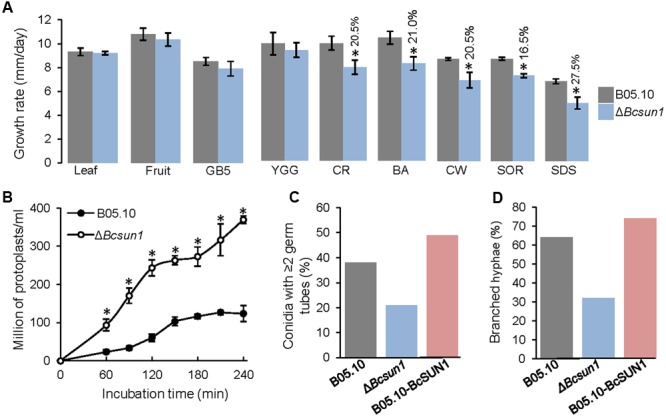
**Role of BcSUN1 in cell wall integrity. (A)** Growth rate of the indicated strains in various media: 6% tomato leaf extract (Leaf), 25% tomato fruit extract (Fruit), minimal medium (GB5), rich medium (YGG), YGG + 0.005% congo red (CR), YGG + 0.4% boric acid (BA), YGG + 0.05% Calcofluor white (CW), YGG + 1 M Sorbitol (SOR), or YGG + 0.02% SDS (SDS). Results are shown as mean ± SD for 9 biological replicates. Asterisks indicate a statistically significant difference (by *t*-test, *p* = 0.05) between the wild type and the mutant in a given medium, and the percentage of reduction in growth rate for the mutant, as compared to the wild type, is indicated above bars. **(B)** Production of protoplasts (mean ± SD, *n* = 3) from the indicated strains when treated with a cocktail of cell wall degrading enzymes. **(C)** Percentage of germinated conidia with two or more germ tubes at 16 h after inoculation in YGG medium (*n* ≥ 100). **(D)** Percentage of hyphae with two or more ramifications at 16 h after inoculation in the same medium (*n* ≥ 100).

Two independent *Bcsun1* knockout mutants (Δ*Bcsun1.1* and Δ*Bcsun1.2*) were generated and characterized by PCR and Southern-blot (Supplementary Figure S1). The two mutants showed no difference with the wild type in the growth rate in rich or minimal media and no difference was found either in media supplemented with tomato leaf or fruit extracts (**Figure 2A**). Nevertheless, the addition of various compounds known to affect the integrity of the plasma membrane or the cell wall integrity did affect the growth rates of the mutant strains (**Figure 2A**). Calcofluor white, congo red, boric acid, and SDS caused a slight, but significant reduction in the growth rate of the Δ*Bcsun1* strains, suggesting that BcSUN1 may have a role in the biogenesis or stability of the cell wall. A smaller growth rate caused by a weaker cell wall can usually be recovered by the addition of osmotic stabilizers to the growth medium. However, in the case of the Δ*Bcsun1* mutants, the presence of an osmotic stabilizer (1 M sorbitol) had a negative effect on growth, and had almost the same impact on the growth rate as CW, CR or boric acid (**Figure 2A**).

Defects in cell wall structure in filamentous fungi can be visualized by testing the sensitivity to protoplast-forming enzymes. With this purpose, young mycelia (conidia germinated for 16 h) were incubated with a cocktail of cell wall degrading enzymes and the production of protoplasts was monitored. The Δ*Bcsun1* mutant strains were more sensitive to these enzymes than the wild type (**Figure 2B**), again pointing to an altered cell wall caused by the deletion of *Bcsun1*. These modifications may also be the reason for the changes detected for the Δ*Bcsun1* mutants in the number of germ tubes per conidium (**Figure 2C**) and in the branching pattern of young hyphae (**Figure 2D**), as an altered cell wall could potentially have an impact on whether or not a new ramification forms at a given moment.

### Deletion of *Bcsun1* Induces Cell Surface Alterations and Modifies the Production of Reproductive Structures

**FIGURE 3:**
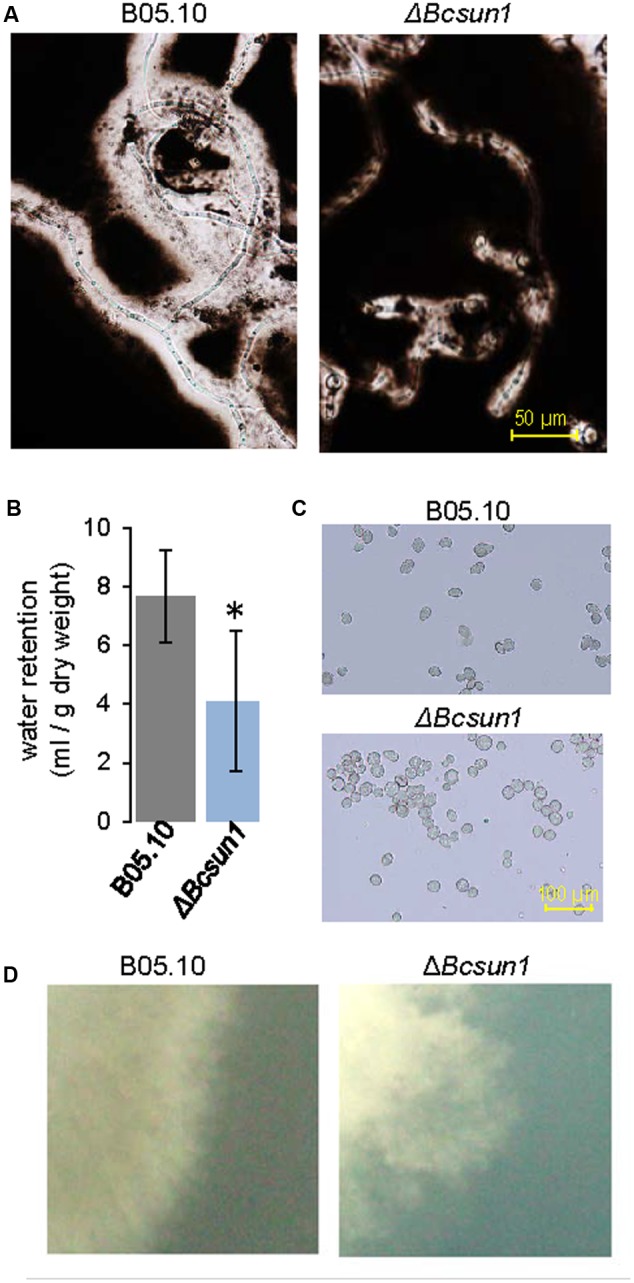
**Lack of BcSUN1 induces cell surface alterations. (A)** ECM displayed by the Δ*Bcsun1* mutant, as compared with the wild type (B05.10), detected by negative India ink staining of conidia germinated in PDB medium for 3 days. **(B)** Water retention capacity (mean ± SD; *n* = 3) of the two strains grown on YGG medium for 3 days. **(C)** Aggregation of Δ*Bcsun1* mutant spores, as compared with the wild type (B05.10). **(D)** Differences in the morphology of the colony borders for the indicated strain when grown in MEA for 3 days.

Although BcSUN1 seems to have a role in the integrity of the cell wall, the protein is also found in the extracellular medium, raising the possibility of its involvement in the structure or metabolism of the ECM. To examine this hypothesis, the Δ*Bcsun1* mutants were grown for 3 days in PDB medium and ECM was negatively stained with India ink (González et al., 2013). ECM is observed as a clear halo surrounding the hyphae against the dark background. The halo was much smaller for the Δ*Bcsun1* strains (**Figure 3A**), as compared with the wild type. One of the functions proposed for the ECM is the retention of water in the cell vicinity (Laspidou and Rittmann, 2002), and a reduced ECM could result in the reduction of the amount of water retained by the mycelium upon filtration of the fungal cultures. Indeed the mycelium of Δ*Bcsun1* strains retained almost 50% less water than the wild type (**Figure 3B**) in agreement with the reduced ECM halo detected by India ink staining (**Figure 3A**).

Additionally, ECM has been proposed to have a role in cell-to-cell attachment (Doss et al., 1995), and therefore the incidence of conidial aggregation was assessed. In the case of the Δ*Bcsun1* mutants most conidia (75%) were found associated to at least one other, while the majority of wild type spores appeared to be in the suspension as individual conidia (**Figure 3C**). Finally, cell surface modifications could provoke differences in the colony morphology, especially at the borders. When fungal strains were grown in MEA medium for 3 days, the mutants showed more irregular and diffuse colony margins as compared to the wild type (**Figure 3D**).

**FIGURE 4:**
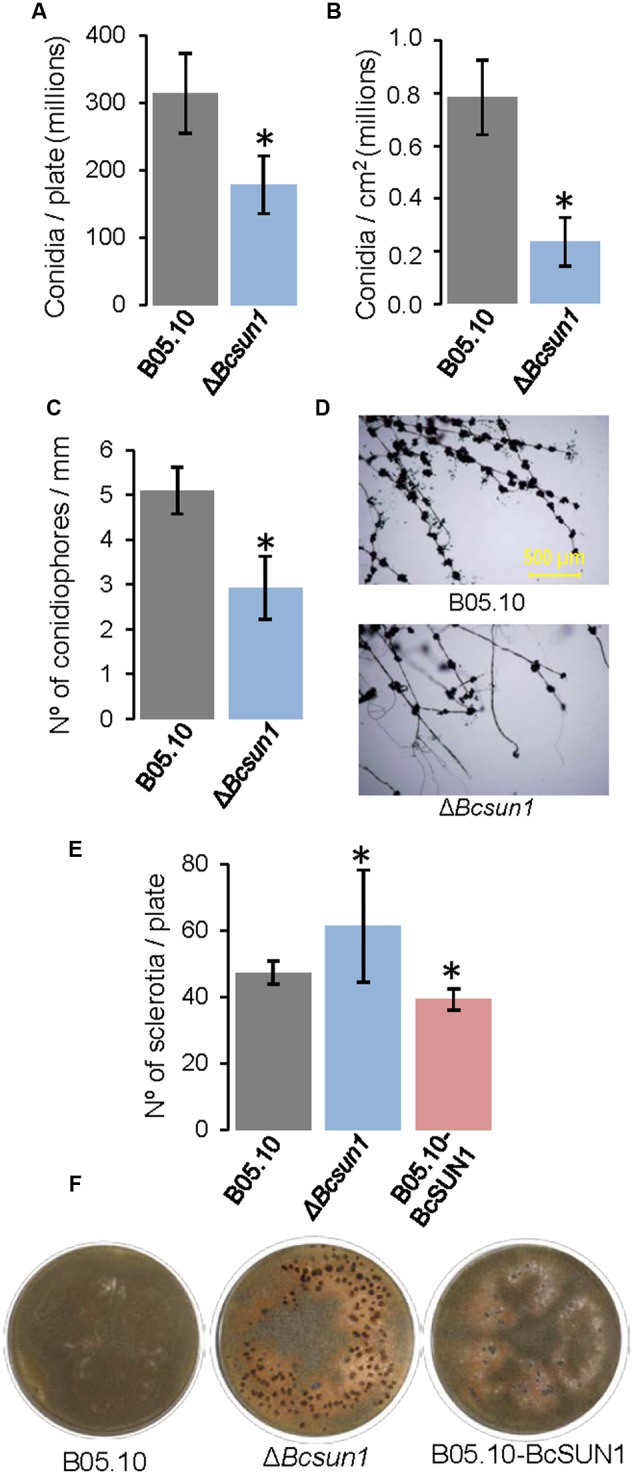
**Production of reproductive structures by the Δ*Bcsun1* mutant. (A)** Amount of conidia produced by the indicated strains in 25% tomato plates growing for 5 days, subjected to 12 h of near-UV light and maintained in the dark for 10 additional days (mean ± SD; *n* = 3). **(B)** Amount of conidia (mean ± SD; *n* = 9) collected from infected tobacco leaves at 10 days after inoculation. **(C)** Number of conidiophores along hyphae (mean ± SD; *n* = 15), produced by the indicated strains growing from YGG-agar plugs over sterile microscope slides after 10 days of incubation. **(D)** Images of conidiophores counted in **(C)**. **(E)** Number of sclerotia produced (mean ± SD; *n* = 3) after 20 days of growth in 25% tomato plates under continuous darkness. **(F)** Images of the plates from which the data in **(E)** were taken. Asterisk on bars indicate a statistically significant difference with the wild type (B05.10).

The influence of the deletion of *Bcsun1* on the production of conidia was assessed determining the number of conidia produced by the two Δ*Bcsun1* mutants, as compared to the wild type (**Figures 4A,B**), but also by studying the number of conidiophores along mature hyphae (**Figures 4C,D**). The results obtained showed a significant reduction in both features for the mutant strains, relative to the wild type. The role of BcSUN1 in the production of sclerotia was also analyzed, and the results showed a significant increase in the number of these survival structures in the mutant strains, as well as a slight reduction in the number of sclerotia for the BcSUN1-overexpressing strain B05.10-BcSUN1 (**Figures 4E,F**). Taken together, these results suggest a role of BcSUN1 in the production of *B. cinerea* reproductive structures.

### BcSUN1 is a Secreted Protein, but is Also Weakly Bound to the Cell Surface

BcSUN1 has previously been identified as a component of *B. cinerea* secretome (Espino et al., 2010; González et al., 2014; González-Fernández et al., 2014) and was more abundant in the extracellular medium when the *O*-glycosylation machinery was altered by mutation of the *Bcpmt1* gene (González et al., 2014). On the other hand, as discussed above, BcSUN1 plays a role in the cell wall. Its *S. cerevisiae* homologues SUN4, UTH1 and SIM1 have been reported to have having multiple cellular locations, either in the cell wall, the extracellular space, or in the mitochondria (Kuznetsov et al., 2013).

**FIGURE 5:**
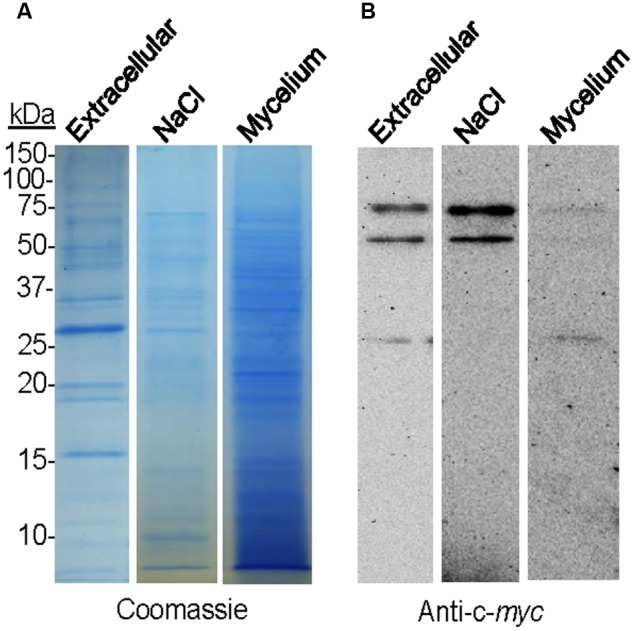
**Localization of BcSUN1. (A)** SDS-PAGE (Coomassie) comparing the amount of total proteins precipitated from the culture medium (Extracellular), extracted with NaCl from the mycelium (NaCl), or extracted from the mycelium with Laemmli sample buffer (Mycelium) in cultures of the B05.10-BcSUN1 strain. The amounts of proteins loaded were those contained in 1 ml of medium (1/30 of total from culture), 0.75 ml of salt-solubilized suspension (1/11 of total from culture), and those extracted from 15 mg of mycelium with Laemmli sample buffer (1/33 of total from culture). **(B)** Western blot (anti-c-*myc*) comparing the amount of recombinant BcSUN1 in the three fractions.

To study the putative binding of BcSUN1 to the cell wall and/or the ECM, the B05.10-BcSUN1 strain, which expressed a version of BcSUN1 bearing a c-*myc* epitope, was grown on YGG-L medium for 16 h and three fractions were recovered representing (i) the soluble extracellular proteins secreted to the culture medium, (ii) the proteins associated to the mycelium that could be released in presence of high salt, and (iii) the proteins extracted from the mycelium with Laemmli sample buffer. Salt treatment can release proteins weakly associated to the fungal cell wall and/or the matrix by van der Waals interactions, hydrogen bonds, and hydrophobic or ionic interactions (Jamet et al., 2008). The same three protein fractions isolated from the wild type strain (B05.10) cultures, as well as proteins precipitated from the un-inoculated medium (YGG-low) were used as negative controls. When proteins were fractionated by SDS-PAGE (**Figure 5A**) and BcSUN1 was visualized by western blot (**Figure 5B**), three different isoforms were found with apparent molecular weights of 25, 50, and 75 kDa, although the expected size for the recombinant polypeptide is 48 kDa. The three isoforms were found in the culture medium fraction, while only the 50 and 75 kDa proteins were released from the mycelium surface by the salt treatment (**Figure 5B**). By considering all the bands obtained in the blots, BcSUN1 was mainly distributed, in almost equal parts, between the extracellular and the salt-extractable fractions (**Figure 5B**). As previously reported (González et al., 2014), no bands were seen in the negative controls (data not shown).

### BcSUN1 is Required for Full Virulence

**FIGURE 6:**
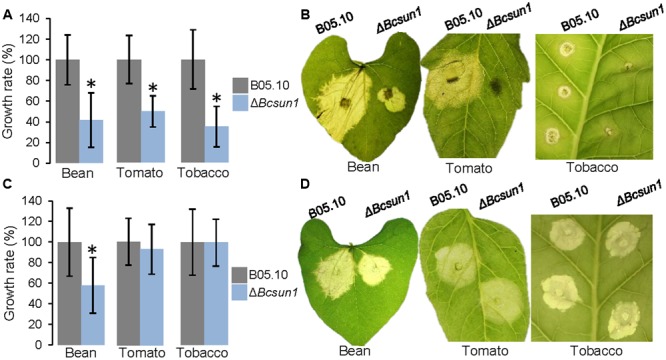
**Infection of different plant hosts with the Δ*Bcsun1* mutants. (A)** Growth rates (mean ± SD; *n* ≥ 10) of the infections caused by the mutant and the wild type on bean, tomato, or tobacco leaves, when inoculated with mycelium plugs. **(B)** Example images of the infections in **(A)** at 5 days after inoculation. **(C)** Same as in **(A)** but in this case inoculations were carried out with 5-μl drops of TGGK containing 5 × 10^6^ conidia/ml. **(D)** Example images of the infections in **(C)** at 5 days after inoculation.

To study the effect of the mutation of *Bcsun1* on fungal virulence, infections were carried out on detached leaves from various plant species. The Δ*Bcsun1* mutants showed, in the first place, a reduction in the number of inoculations actually producing a spreading lesion when tomato or tobacco leaves were inoculated with agar plugs (**Table 2**). Such a reduction in the proportion of expanding lesions, however, was not observed in the case of bean leaves inoculated with agar plugs, or in any host plant tested when inoculations were carried out with conidia. When the growth rate of the expanding lesions was measured, a significant reduction was observed for the Δ*Bcsun1* mutants in the three host plant species when inoculations were carried out with agar plugs (**Figure 6**). In the case of inoculations with conidia, however, a significant reduction in expanding lesion growth rate was observed for the Δ*Bcsun1* mutants only on bean leaves, while lesions on tomato and tobacco leaves spread at the same rate for the mutants and the wild type. In all these tests, no significant differences were found for the B05.10-BcSUN1 strain, as compared to the wild type (not shown).

**Table 2:** Percentage of inoculations with the Δ*Bcsun1* mutants resulting in spreading infections.

	Bean leaves (*n* = 15)		Tomato leaves (*n* = 16)		Tobacco leaves (*n* ≥ 19)	
	Plugs	Conidia	Plugs	Conidia	Plugs	Conidia
B05.10	100	100	86.7	100	91,7	100
Δ*Bcsun1.1*	100	100	66.7	100	47.4	100
Δ*Bcsun1.2*	100	100	66.7	100	30.0	100
B05.10-BcSUN1	100	100	100	100	89.5	100

*The indicated plant leaves were inoculated with either agar plugs or conidia, and the generation of spreading lesions was recorded visually during the first 3 days after inoculation.*

**FIGURE 7:**
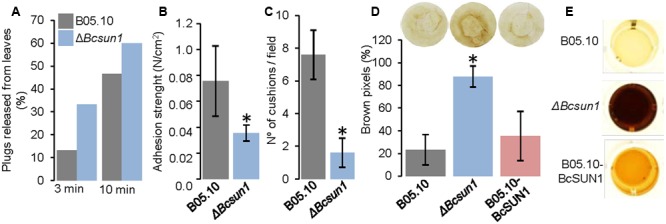
**Surface interaction between knockout *Bcsun1* mutants and plant host. (A)** Percentage of plugs released from tobacco leaves by washing with water (for the times indicated at the bottom) at 24 h after inoculation (*n* ≥ 15). **(B)** Adhesion strength of mycelium plugs to tobacco leaves after 24 h of incubation (mean ± SD; *n* = 15). **(C)** Number of infection cushions produced by the indicated strains from agar plugs on glass slides. The data correspond to the average number of cushion determined from 10 microscopic fields randomly chosen. **(D)** Production of H_2_O_2_ (as detected with DAB) in infected tobacco leaves, 36 h after inoculation with conidia of the indicated strains. Upper panel shows an example of the results, and lower panel shows a semi-quantification of H_2_O_2_ produced, expressed as the percentage of brown pixels in a circumference of constant area (calculated with the software Fiji, mean ± SD; *n* = 5). **(E)** Secretion of H_2_O_2_ by the indicated strains grown for 4 days in YGG medium, detected with DAB.

The initial stage of the infection process is characterized by adhesion of hyphae to the host surface. To analyze if the adherence of fungal hyphae to the plant surface is modified by *Bcsun1* deletion, the adhesion of mutant strains to host tissue was examined by measuring the percentage of mycelium plugs detached from the surface of tobacco leaves after washing with water at 24 h post inoculation (**Figure 7A**), and by measuring the physical force that was necessary to detach individual plugs (**Figure 7B**). The Δ*Bcsun1* strains showed both an increase in the percentage of plugs that could be detached by washing and a decrease of the average adhesion force of individual plugs to the plant surface. Additionally, a microscopic analysis was done to examine if the deletion of the *Bcsun1* gene induced changes in the number and/or the structure of infection cushions. The morphology of the cushions produced by the Δ*Bcsun1* mutants was similar to those generated by the wild type or the B05.10-BcSUN1 strains (data not shown). However, the number of infection cushions was reduced between 50 to 70% in the mutants as compared to the wild type (**Figure 7C**).

Finally, the production of ROS in tobacco leaves during infection was analyzed, as it has been reported that UTH1 from *S. cerevisiae* and UvSUN2 from *U. virens* have a role in oxidative-stress response (Bandara et al., 1998; Yu et al., 2015). An increase in the reddish-brown DAB precipitate was found in the lesions caused by the Δ*Bcsun1* strains, which was almost fourfold more intense than that produced by the wild type strain (**Figure 7D**). An increase in ROS production by the Δ*Bcsun1* mutants was also observed *in vitro* using a semi-quantitative method (**Figure 7E**). Furthermore, the addition of hydrogen peroxide to the culture medium reduced the growth rate of the Δ*Bcsun1* strains (by 14%), indicating that ROS sensitivity seems also to be affected. All these changes in ROS production and tolerance may have a role in contributing to the lower virulence of the Δ*Bcsun1* mutants.

## Discussion

### The *B. cinerea* Genome Encodes Two SUN-Family Proteins

β-Glucosidase SUN family members have been extensively studied in yeast and have been linked to diverse cellular functions such as cell wall biogenesis and septation, mitochondrial biogenesis, aging and DNA replication (Camougrand et al., 2000; Mouassite et al., 2000a; Velours et al., 2002; Hiller et al., 2007; Kuznetsov et al., 2013). BcSUN1 is a glycoprotein secreted by *B. cinerea* (Espino et al., 2010; González et al., 2014) that belongs to the Group-I of the SUN family, showing the highly conserved *C*-terminal region characteristic for this group of proteins. The search for other members of Group-I in the *B. cinerea* genome confirmed BcSUN1 as the single protein belonging to this group, but it revealed the existence of the Bcin07g06600.1 gene, which encodes a secreted protein of 530 amino acids with high homology with YMR244W from *S. cerevisiae* and Adg3 from *S. pombe* (Firon et al., 2007), both members of Group-II of the SUN family. This second member of the SUN family has been predicted to be GPI-anchored to the cell wall (de Groot et al., 2013) and to be highly *O*-glycosylated (González et al., 2012), features that are typical for Group-II proteins (Firon et al., 2007; Gastebois et al., 2013). The presence of a single protein from each group of the SUN family is considered characteristic for euascomycetes (Firon et al., 2007; de Groot et al., 2009; de Groot et al., 2013; Gastebois et al., 2013), while in yeasts the number of Group-I members can mount up to four, as in *S. cerevisiae* (Firon et al., 2007).

SUN family proteins have barely been experimentally characterized in filamentous fungi. AfSUN1 from *A. fumigatus* is the only Group-I protein studied so far, and it was shown to have a unique hydrolytic activity on β-1,3-glucan (Gastebois et al., 2013) which has resulted in classifying it in a new CAZY class, GH132. The high similarity of BcSUN1 to AfSUN1 suggests that the *B. cinerea* protein may also display this enzymatic activity. However, our attempts to purify BcSUN1 from the strain B05.10-BcSUN1, in order to confirm its enzymatic activity, were unsuccessful.

### BcSUN1 Plays a Key Role in Maintaining the Cell Wall and Extracellular Matrix

Previous analysis of *B. cinerea* secretomes obtained under different growth conditions, revealed BcSUN1 as a protein secreted by old mycelium grown on minimal and rich media (González et al., 2014; González-Fernández et al., 2014), but also as a member of the early secretome (Espino et al., 2010). Transcriptional analysis of conidial germination on wax-coated surfaces showed that *Bcsun1* expression was induced already in the first hour of conidial germination, and its level remained constant at least for the first 15 h after inoculation (Leroch et al., 2013). In the present work, we corroborated the expression of the *Bcsun1* gene at the early stages after inoculation, both in axenic culture and *in planta*, and observed a continuous increase as the infection progresses, a pattern clearly consistent with a role in the morphogenesis and mycelial growth, as has been described for AfSUN1 (Gastebois et al., 2013).

The use of cell wall perturbing agents caused a reduction in the growth rates of Δ*Bcsun1* mutants (**Figure 2A**). This was the case for congo red, which blocks lateral interaction between glucan chains causing loss of cell wall rigidity (Kopecka and Gabriel, 1992; Ram and Klis, 2006), and Calcofluor white, which binds to chitin and interferes with its polymerization preventing the interactions between chitin and glucans (Roncero et al., 1988; Ram and Klis, 2006). Δ*Bcsun1* mutants also grew poorly in medium supplemented with boric acid (**Figure 2A**), which in *C. albicans* suppresses hyphal growth (De Seta et al., 2009) and in *S. cerevisiae* leads to the synthesis of irregular cell wall protuberances and the formation of irregular, chitin-rich septa (Schmidt et al., 2010). Finally, growth on high levels of sorbitol also resulted in growth reduction of the Δ*Bcsun1* mutants (**Figure 2A**). This sugar alcohol is used frequently to stabilize osmotically damaged cell walls, but it may also increase glycerol production causing a reduction in the synthesis of cell wall components (Gorka-Niec et al., 2010). These findings strongly suggest that BcSUN1 may play a key role in the metabolism of the cell wall, since its absence in the mutants results in weaker walls. Additional evidence comes from the increased sensitivity of the mutant strains to SDS (**Figure 2A**), an anionic detergent that induces lysis of cells with fragile cell walls (Shimizu et al., 1994), and from the higher sensitivity to protoplast-forming lytic enzymes (**Figure 2B**). Moreover, the changes in the hyphal branching pattern observed for the Δ*Bcsun1* mutants (**Figures 2C,D**) also suggest modifications in cell septation and cell wall remodeling.

A role of SUN family proteins in cell wall biogenesis has also been described in other organisms. In the yeast *S. cerevisiae*, the four *S. cerevisiae* proteins act by remodeling the cell wall (Kuznetsov et al., 2013). In fact, knockout mutants in the UTH1 gene showed similar sensitivity to cell wall modifying substances (Kuznetsov et al., 2013) as reported here for Δ*Bcsun1* mutants, and the reduction in the number of protoplasts generated from the UTH1 mutant strain has been related with an increase in β-1,6-glucan and chitin composition of the yeast cell wall (Ritch et al., 2010). SUN41 from *C. albicans* is also involved in morphogenesis, cell wall biogenesis and is necessary during yeast branching (Hiller et al., 2007). WMSU1 from *Williopsis saturnus* has been reported to be involved in cell wall metabolism (Guyard et al., 2000). Finally, PSU1 from *S. pombe* plays a critical role in cell separation (Omi et al., 1999; de Groot et al., 2007). In filamentous fungi, the deletion of AfSUN1 also caused alterations on the mycelium growth and hyphal morphogenesis, although no differences were reported/observed in the cell wall composition of the mutant strain (Gastebois et al., 2013).

*Botrytis cinerea* produces a prominent ECM involved in the adhesion to the host tissues (Doss, 1999; Cooper et al., 2000; Gil-ad et al., 2002; Doss et al., 2003). ECM was greatly reduced in Δ*Bcsun1* strains (**Figure 3A**), which resulted in a reduced capacity to retain water (**Figure 3B**). This may be a consequence of a reduced or weaker cell wall, since an altered glucan-chitin network may have less potential sites for binding to or interaction with the ECM components, but BcSUN1 itself may also be relevant for linking ECM components. In this context, it is interesting that the SUN41 protein from *C. albicans* has been proposed to play an important role in biofilm formation (Hiller et al., 2007; Norice et al., 2007).

The alterations in the Δ*Bcsun1* mutants regarding conidia and sclerotia production, conidia aggregation (**Figure 3C**), or colony morphology, may all be consequences of an altered cell wall and/or ECM. The aggregation of *A. fumigatus* germinating conidia, for example, is dependent on cell wall α-1,3-glucans and may be prevented by the addition of α-1,3-glucanase (Fontaine et al., 2010), and similar phenotypic features have been reported for a *B. cinerea* mutant in the gene *Bcpmr1* displaying an altered cell wall (Plaza et al., 2015).

### BcSUN1 is Associated with the Cell Surface and is Also Secreted

The c-*myc*-tagged version of BcSUN1 was identified in the secretome of the B05.10-BcSUN1 strain in three isoforms (**Figure 5B**), with molecular weights that differ from the expected size for the recombinant protein (González et al., 2014). Glycosylation may contribute to this heterogeneity, as not only 74 *O*-glycosylation sites are predicted for the protein (González et al., 2012), but it has also been experimentally shown to contain mannose residues linked by α1-2 or α1-3 glycosidic bonds (González et al., 2014). Additionally, the present study detected BcSUN1 both in the extracellular media as a soluble protein and also associated to the cell wall (**Figure 5**). Such dual localization has also been described for UTH1, SUN4 and SIM1 (Kuznetsov et al., 2013), and similar findings were reported for *C. albicans* SUN41 and SUN42 proteins (Hiller et al., 2007; Sorgo et al., 2010). The *S. pombe* psu1 protein, however, is covalently bound to the glucan network of the cell wall via a mild alkali-sensitive phosphodiester bridge (de Groot et al., 2007). The cell-wall association of BcSUN1 is consistent with a role in cell wall metabolism, as discussed above, and it may play a role there in remodeling the extracellular structures including cell wall and ECM.

### BcSUN1 is a Virulence Factor

*Bcsun1* is expressed from the very early stage of fungus-plant interaction, and *Bcsun1* mRNA levels increase as the lesions become necrotic (**Figure 1B**). Previously, Smith et al. (2014) identified *Bcsun1* as one of the genes induced *in planta* during infection of *Solanum lycopersicoides* leaves. Altogether, these results suggested a role of BcSUN1 in fungal pathogenesis. Indeed BcSUN1 is involved in the adhesion of the mycelium to the host surface during infection (**Figure 7**), most probably by altering the properties of the ECM and thus changing its adhesive capacity. The chemical nature of these alterations remains to be investigated. The reduced adherence of the Δ*Bcsun1* mutants could explain the lower capacity of the mutants to initiate a successful infection (**Table 2**), and this effect may be more prominent in natural infections in the field, where a single conidium landing on the plant surface is the predominant source of inoculum. We also found that *Bcsun1* mutants showed a reduced production of infection cushions (**Figure 7C**), which are specialized, dense and highly branched structures that play a critical role in mycelium-derived infections (Choquer et al., 2007), as well as an overall reduced virulence on all plant hosts tested (**Figure 6**). Moreover, Δ*Bcsun1* mutants are affected in the production of conidia and sclerotia (**Figure 4**), which may in turn affect the dispersal of the pathogen. Finally, the Δ*Bcsun1* mutants showed an enhanced production of hydrogen peroxide in axenic culture and *in planta* (**Figure 7**), which may also contribute to their altered virulence. In conclusion, we report here for the first time the involvement of a protein from the β-glucosidase SUN family in the virulence of a phytopathogenic fungus. Since this family has been found only in ascomycetes, these proteins represent a promising novel target to develop new control strategies against *B. cinerea.*

## Author Contributions

All authors participated in the design of the experiments as well as the analysis/evaluation of the results. AP-H and MG drafted the initial manuscript and all authors participated in the editing and approved its final version.

## Conflict of Interest Statement

The authors declare that the research was conducted in the absence of any commercial or financial relationships that could be construed as a potential conflict of interest.

The reviewer AMR and handling Editor declared their shared affiliation, and the handling Editor states that the process nevertheless met the standards of a fair and objective review.

## Footnotes

1 http://fungi.ensembl.org/Botrytis_cinerea
