# Supplementary material for: BcSUN1, a B. cinerea SUN-Family Protein, Is Involved in Virulence
Source: Front Microbiol. 2017 Jan 20;8:35. doi: 10.3389/fmicb.2017.00035 (PMC5247446; doi:10.3389/fmicb.2017.00035)
Supplement: Supplementary file 1 [file Presentation_1.PDF]

Supplementary Material

BcSUN1contributes to *Botrytis cinerea* cell wall and extracellular matrix integrity, and is involved in virulence

Alicia Pérez-Hernández, Mario González, Celedonio González, Jan A. L. van Kan and Nélida Brito*

*** Correspondence:** Dr. Nélida Brito; nbrito@ull.edu.es

Supplementary Figures

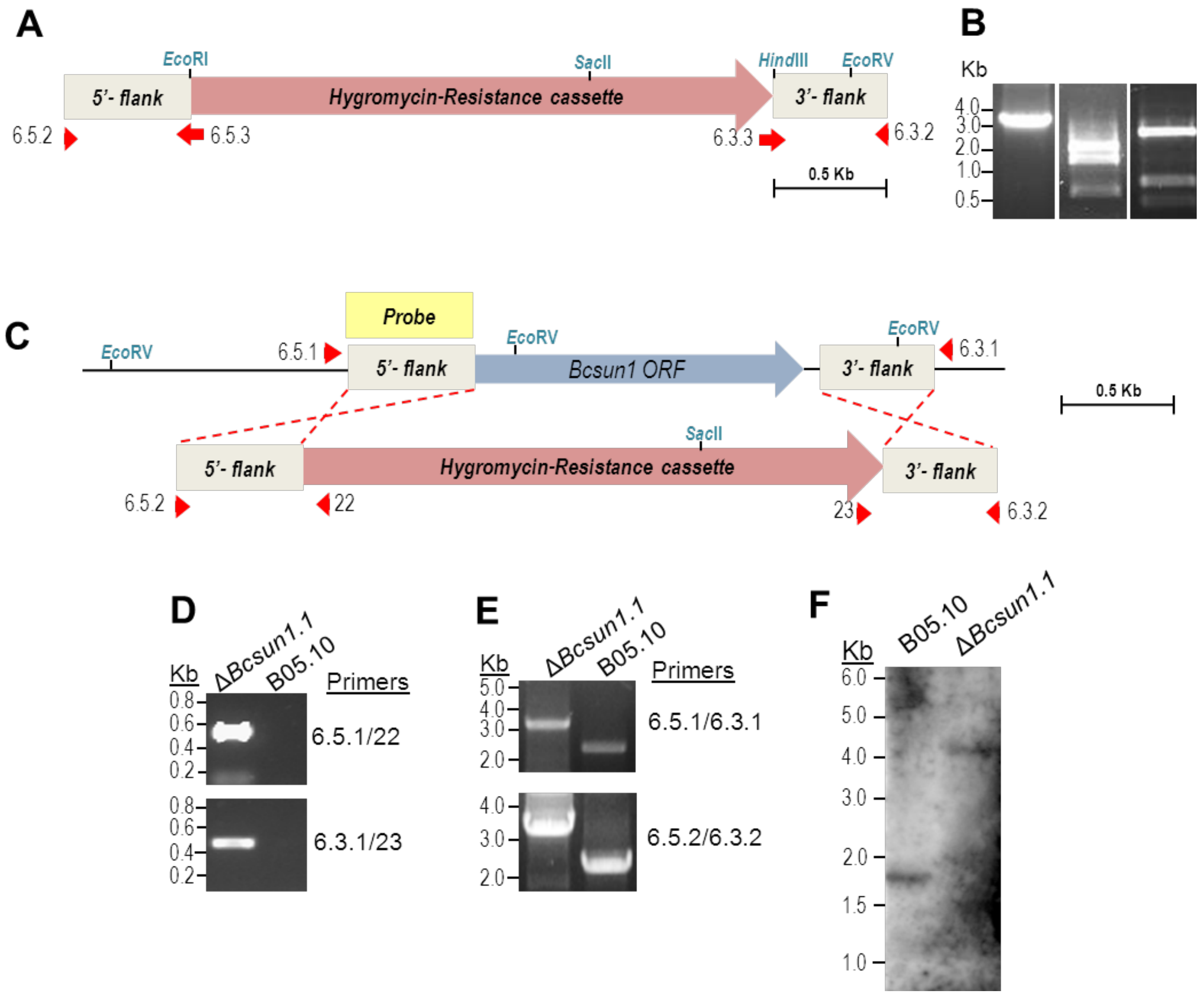

Supplementary Figure S1. Strategy used for the generation of *Bcsun1* knockout mutants. (A) Generation of the *Bcsun1* replacement cassette by Overlap Extension PCR. The 5'-flank region of *Bcsun1* was amplified with primers 6.5.2/6.5.3, and the 3'-flank region with primers 6.3.2/6.3.3, using *B. cinerea* B05.10 genomic DNA as template. Primers 6.5.3 and 6.3.3 have tails complementary to the ends of the hygromycin resistance cassette (RC), which was obtained from pLOB7 by digestion with *Eco*RI and *Hind*III. (B) Agarose gel electrophoresis showing the RC (3.4 Kb) as well as the fragments resulting from its digestion with the indicated enzymes. (C) Double homologous recombination (red dotted lines) of the RC at the *Bcsun1* locus resulted in generation of the Δ*Bcsun1.1* and Δ*Bcsun1.2* mutants. Note that primers 6.5.1 and 6.3.1 bind to genomic DNA outside the homologous recombination region. (D) PCR products obtained with the indicated primer pairs and genomic DNA from the Δ*Bcsun1.1* mutant or the wild type (B05.10), showing the correct integration of the RC in the mutant. (E) PCR products obtained with the indicated primers to confirm the absence of the *Bcsun1* ORF (2.4 Kb) and the presence of the RC (3.4 Kb) in the mutant. (F) Southern-blot of *Eco*RV-digested genomic DNA probed with the 5'-flank region of *Bcsun1* (probe). Results showed the wild-type 1.7 Kb fragment and the single 4.2-Kb fragment expected in the mutant. No additional bands were observed, indicating the absence of additional non-homologous integrations of the RC. Similar results were obtained when genomic DNA from the Δ*Bcsun1.2* mutant was used (not shown).

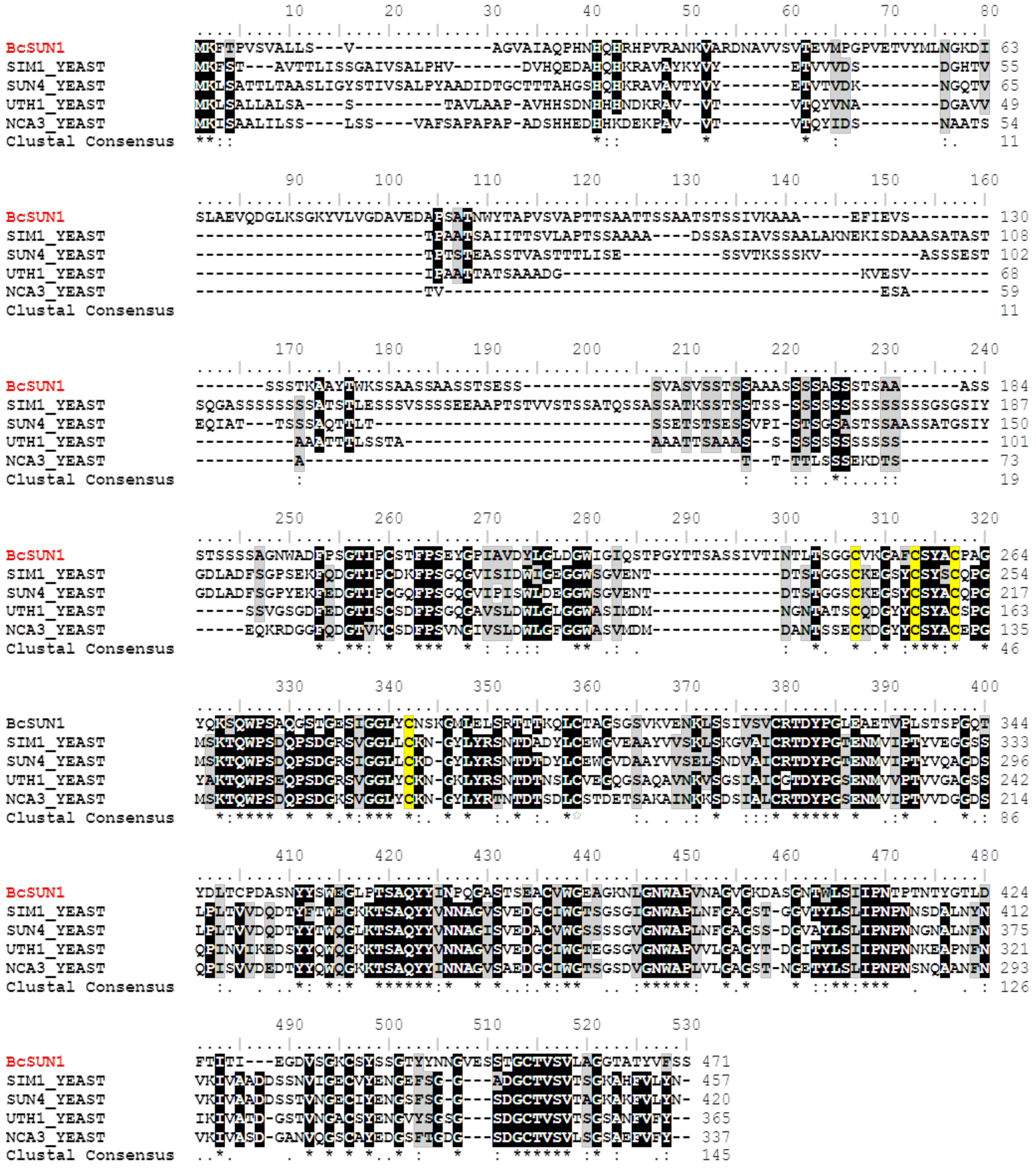

Supplementary Figure S2. Alignment of BcSUN1 with the four *S. cerevisiae* proteins of Group-I of the SUN family. Identical or similar residues are shown with black or gray background, respectively. The four cysteine residues of the putative Fe-binding domain (Cys-X_5-Cys-X_3-Cys-X_{24}-Cys) are marked in yellow. Protein accession numbers are as follows: P40472 (SIM1_YEAST), P36135 (UTH1_YEAST), P32493 (NCA3_YEAST) and P53616 (SUN4_YEAST).

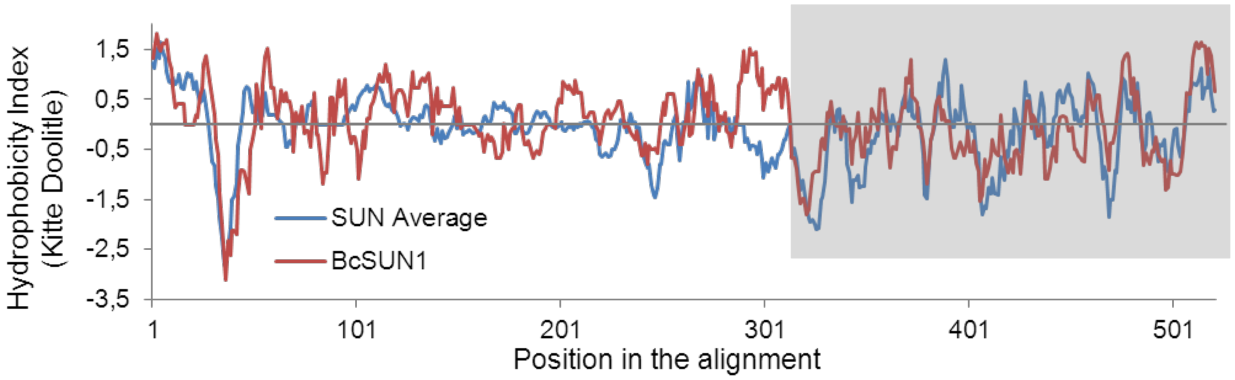

Supplementary Figure S3. Hydropathy profiles of BcSUN1 and the four *S. cerevisiae* proteins of Group-I of the SUN family. The average hydropathy profile for SIM1, UTH1, NCA3 and SUN4 (blue line) was calculated from their alignment and is compared to profile of BcSUN1. A grey background highlights the strong similarity in the *C*-terminal regions.

Supplementary Table

Supplementary Table S1. Primers used in this study

Primer name	Sequence (5’ → 3’)	Target	Comments
**SUN1_RT_FW**	TGGTGTCGGCAAAGATGC	*Bcsun1*	Amplification of a 192-bp fragment from *Bcsun1* in Q-RT-PCR experiments
**SUN1_RT_RV**	TGCGAGAACAGAGACGGTG		
**ACTAFW**	GGTAACATTGTTATGTCTGG	*actA*	Amplification of a 100-bp fragment from *actA* gene in Q-RT-PCR experiments
**ACTARV**	CGTCGATGAAGGTCAAG		
**6.5.2**	GCCACTTTTATCTCAGCGCC	*Bcsun1*	Amplification of 5’flank fragment of *Bcsun1* for the generation of the replacement cassette and characterization of mutants
**6.5.3**	CCCCGGGTACCGAGCTCGAATTC<br>ACGCTGAGAAGCGCAACAG	hygromycin resistance cassette/*Bcsun1*	Amplification of 5’flank fragment of *Bcsun1* for the generation of the replacement cassette. Tail complementary to hygromycin resistant cassette is underlined in the sequence.
**6.3.2**	GAGTTAACCAAGGTGATCGC	*Bcsun1*	Amplification of 3’flank fragment of *Bcsun1* for the generation of the replacement cassette and characterization of mutants
**6.3.3**	AACTCGGCGCGCCGAAGCTTATC<br>ATAGGCGCTTGAGTCTC	hygromycin resistance cassette/*Bcsun1*	Amplification of 3’flank fragment of *Bcsun1* for the generation of the replacement cassette. Tail complementary to hygromycin resistant cassette is underlined in the sequence
**6.5.1**	CACACGAAAATAAACAGC	*Bcsun1*	Characterization of *Bcsun1* mutants
**23**	GTAACCATGCATGGTTGCCT	hygromycin resistance cassette	
**6.3.1**	GCACTCTGTACCAGGAGC	*Bcsun1*	Characterization of *Bcsun1* mutants
**22**	GGGTACCGAGCTCGAATTC	hygromycin resistance cassette	
